# Congenital heart disease combined with Arrhythmogenic Right Ventricular Cardiomyopathy: A CARE compliant case report and literature review

**DOI:** 10.1097/MD.0000000000020279

**Published:** 2020-06-19

**Authors:** Chutong Ren, Zhenfei Fang, Yanshu Zhao, Jun Luo

**Affiliations:** a Department of Minimal Invasive Surgery; b Department of Cardiology, The Second Xiangya Hospital, Central South University, Changsha, Hunan Province, China.

**Keywords:** arrhythmogenic right ventricular cardiomyopathy, atrial septal defect, congenital heart disease

## Abstract

**Rationale:**

Arrhythmogenic right ventricular cardiomyopathy (ARVC) is a hereditary cardiomyopathy disease discovered in 1994. Though there are advances in diagnosis of arrhythmogenic right ventricular cardiomyopathy, early diagnosis is still difficult especially when it is combined with other diseases with similar pathophysiologic changes, such as left to right shunt congenital heart disease (CHD). In this paper, we reported a case of CHD combined with ARVC.

**Patient concerns:**

The patient was referred to us for chest tightness and shortness of breath after physical activities. His cardiac MRI indicated the possibility of arrhythmogenic right ventricular cardiomyopathy. He was diagnosed with a large atrial septal defect (ASD) through ultrasound examination.

**Diagnosis:**

CHD ASD combined with arrhythmogenic right ventricular cardiomyopathy.

**Interventions:**

The patient underwent occlusion of the ASD and he was followed-up closely. His symptoms were relieved a lot and the activity tolerance was elevated.

**Lessons:**

CHD may accompany with arrhythmogenic right ventricular cardiomyopathy. Careful history collection and comprehensive examinations should be emphasized. We firmly believe that our work will be helpful for the medical practice of similar complicated cardiovascular diseases.

## Abstract 2

Supplemental Digital Content is available in the text

**Established Facts and Novel Insights**

Established Facts:

1. Arrhythmogenic right ventricular cardiomyopathy (ARVC) is a hereditary disease usually causing sudden cardiac death in youth, which indicates the importance of diagnosis in its early stage.
2. According to the pathological and physiological changes caused by ARVC, ARVC tends to be confusing when it combines with some diseases that may also lead to right ventricular enlargement, making the diagnosis more challenging.

Novel Insights:

1. ARVC can accompany with congenital heart disease; in such occasion, careful differential diagnoses are required.
2. Only 5 cases were reported as congenital heart disease combined with ARVC; so the summary of the clinical features of these cases may provide a great reference for further practice.

## 1 Introduction

Arrhythmogenic right ventricular cardiomyopathy (ARVC) is a family disease related to syncope and sudden cardiac death. It has an estimated prevalence of 1 in 5000 in the general population^[1]^ and characterized by ventricular arrhythmia with left bundle branch block and progressive fibrofatty infiltrations of the right ventricle. The diagnosis of ARVC is rather challenging, especially in the early stage. Here, we present an interesting case of a 58-year-old male patient who was referred to us for chest tightness and shortness of breath after physical activities. After careful examination and evaluation, he was diagnosed with the atrial septal defect (ASD) while his clinical features also met the diagnosis criteria of ARVC. According to the literature review, only 5 cases (including the present case) were reported as congenital heart disease (CHD) combined with ARVC. Thus, we summarized these cases here, hoping to provide more data for further clinical practice regarding to these two diseases.

## 2 Case presentation

### 2.1 Clinical history

A 58-year-old male was referred to the hospital for chest tightness and shortness of breath after physical activities for over 40 years. His symptoms were exacerbated for 1 month. Since the patient was a 10-year-old boy, he showed decreased exercise capacity compared to his peers. He could only tolerate 200 meters’ run before symptoms such as chest tightness, shortness of breath and palpitation appeared. Amaurosis sometimes appeared after physical activities but these symptoms could relieve after rest. Since 8 years ago, the patient could not tolerate 4 to 5 floor climbing. And this year, he had chest tightness and shortness of breath after only 2-floor climbing, accompanied by precardium area pain radiating to the back and shoulders. He could not lie down at night and had edema, abdominal distension, weakness and loss of appetite. The patient had a history of chronic bronchitis which was well controlled. He had been smoking for over 30 years. The family history of the patient deserves to be mentioned. His mother died of a sudden heart attack at about 50 years old. And his daughter was diagnosed with ventricular septal defect (3 mm in diameter) during the antenatal examination. His physical examination results were listed as follow. The left boundary of his heart showed expansion. Cardiac auscultation showed arrhythmia and blowing systolic murmur at 2nd-3rd intercostal space at the left margin of the sternum. Pulse deficit was obvious. No other positive signs were found.

### 2.2 Accessory examination

A series of examinations were carried out after the patient was admitted to the ward. His 24-hour Holter showed that:

(1) atrial fibrillation with slightly slow mean heart rate,
(2) frequent multi-source premature ventricular contractions, part of which were bigeminal and part paired,
(3) occasional ventricular extrasystole,
(4) complete right bundle branch block,
(5) occasional change of T waves in partial lead connection.

**Figure 1:**
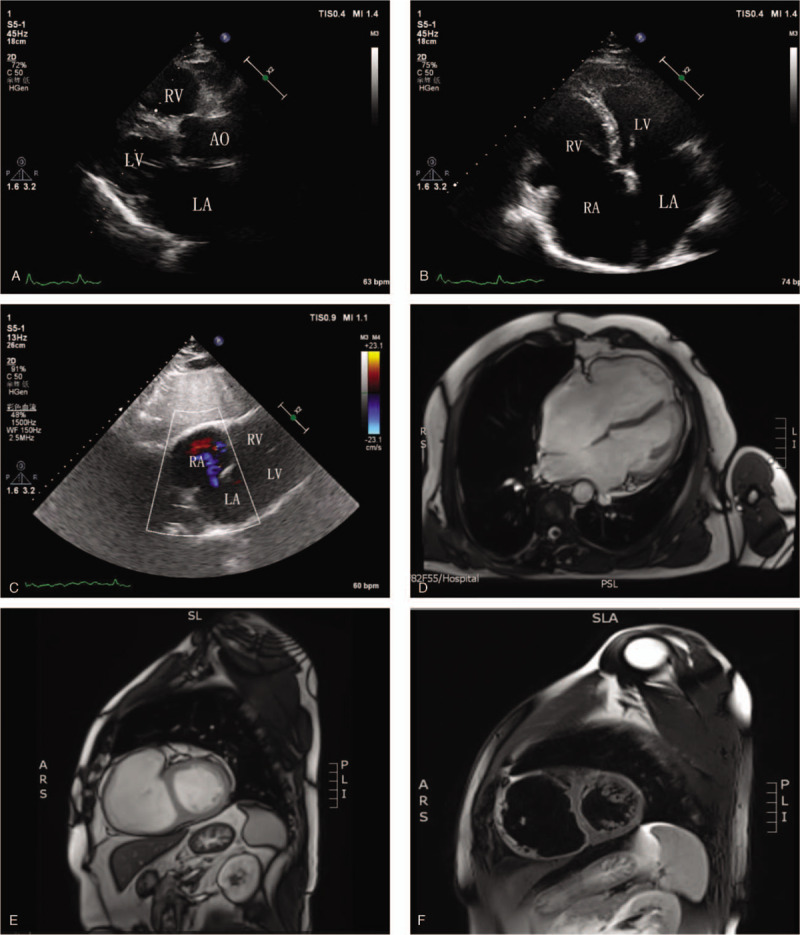
The echocardiography of the patient. (A) The parasternal long axis view; (B) The apical 4 chamber view; (C) The subxiphoid 4 chamber view. The cardiac magnetic resonance imaging of the patient which shows typical change of ARVC (D, E, F). ARVC = arrhythmogenic right ventricular cardiomyopathy.

The chest radiography showed an expanded heart boundary, which coincided with the physical examination. He also had an echocardiography examination (Fig. 1A–C), which shows:

(1) ASD (secondary orifice, left to right shunt), about 25.7 mm in diameter,
(2) Enlargement of right atrium, right ventricle and left atrium, slightly thin right ventricular wall and irregular shape of the right ventricle,
(3) uncoordinated ventricular wall movement,
(4) slightly wider ascending aorta,
(5) slight reflux in the mitral valve, tricuspid valve and pulmonary valve area,
(6) Arrhythmia,
(7) normal systolic function of left ventricular and small decrease in the function of right-side heart (EF was only for reference because of atrial fibrillation).

**Figure 2:**
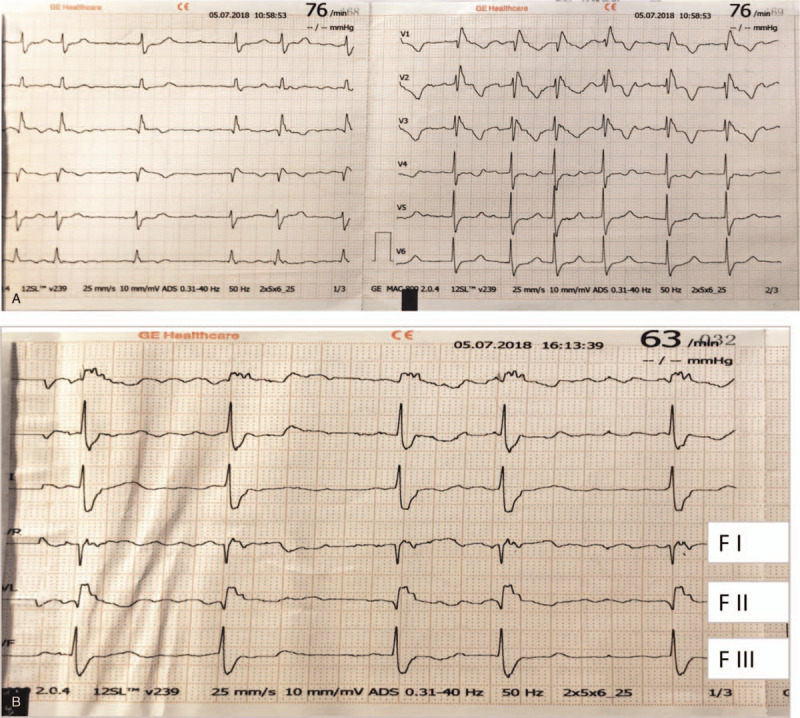
The ECG of the patient. (A) Routine 12-leads electrocardiogram; (B) Fontaine lead ECG which showed typical epsilon wave. ECG = electrocardiogram.

His cardiac MRI (Fig. 1D–F) showed the possibility of ARVC. The routine 12-leads electrocardiogram of this patient is showed in Fig. 2A. For the purpose of proving the MRI diagnosis, a fontaine lead electrocardiogram was performed in order to show right ventricular myocardial depolarization better. Consequently, a typical epsilon wave was detected (Fig. 2B). In addition, his blood pressure pro-BNP level was elevated evidently. The gene detection related to ARVC was advised to the patient's family. However, his family refused and chose to continue the follow-up.

### 2.3 Therapeutic regimen

After careful consideration, we advised the patients to undergo occlusion of ASD surgery. The patient underwent this surgery and his symptoms were relieved slightly after the surgery. The follow-up was of vital importance for the patient. In addition, gene detection should still be carried out if necessary during further consultation.

### 2.4 Follow-up

**Figure 3:**
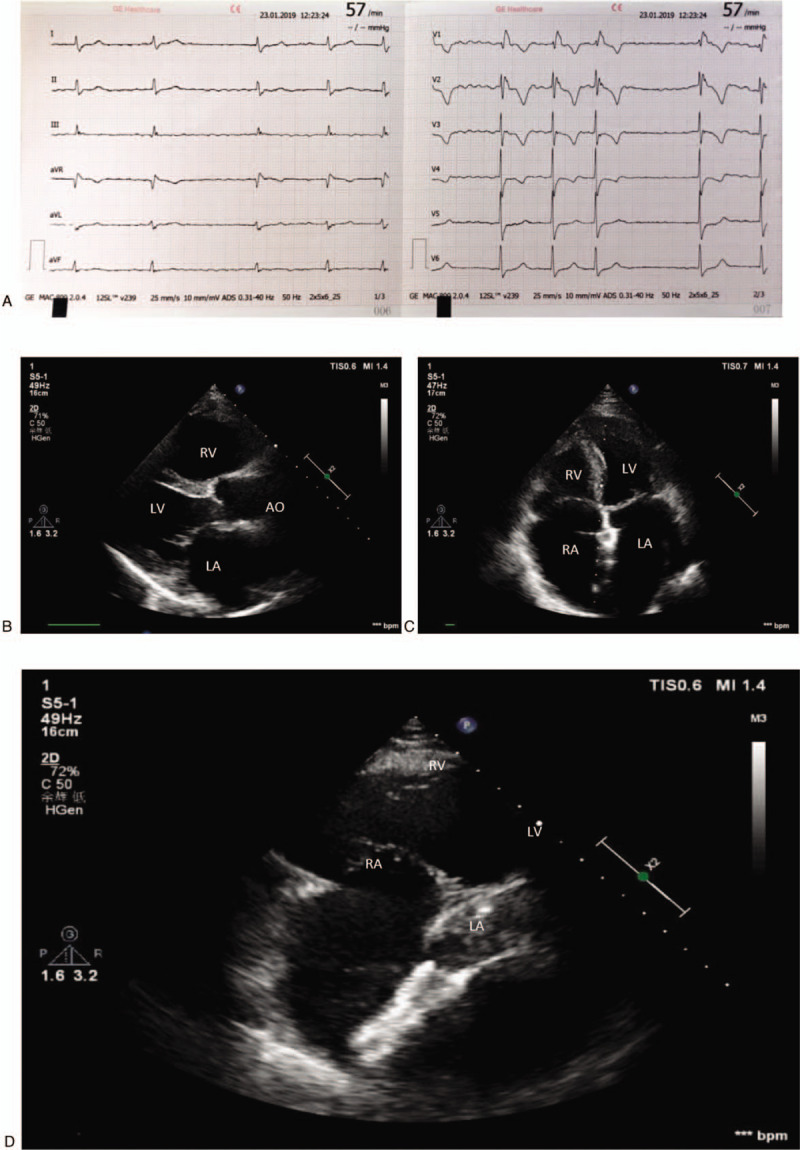
The follow-up examination of the patient 6 mo after surgery. (A) Routine 12-leads electrocardiogram; (B) The parasternal long axis view echocardiography; (C) The apical 4 chamber view echocardiography; (D) The subxiphoid 4 chamber view echocardiography.

The patient went back to the clinic for his reexamination 6 months after the surgery. His symptoms were relieved a lot and his exercise tolerance increased evidently. The patient's blood pressure pro-BNP level decreased to be normal. The 12-lead electrocardiograph showed atrial fibrillation with a low ventricular rate, incomplete right bundle branch block and abnormal T wave (Fig. 3A). The echocardiography showed the decreased cardiac size and effective occlusion (Fig. 3B–D).

## 3 Discussion

ARVC is a type of inherited cardiomyopathy characterized by ventricular arrhythmia with left bundle branch block and progressive fibrofatty infiltrations of right ventricle.^[2]^ Life-threatening ventricular arrhythmia is very common for ARVC patients and it usually leads to sudden cardiac death.^[3]^ The diagnosis of ARVC is rather complicated. According to the 2010 Revised Task Force Criteria for the diagnosis of ARVC as listed in Table 1, it is based on structural alternations, histologic character, Electrocardiograph, arrhythmic, and familial features.^[4]^ Although the new diagnosis material is more sensitive than the original one in 1994, the diagnosis of ARVC is still challenging, especially for the patients in the early stage of disease^[5]^ and it cannot differentiate cardiac sarcoidosis from ARVC.^[6]^

**Table 1:**
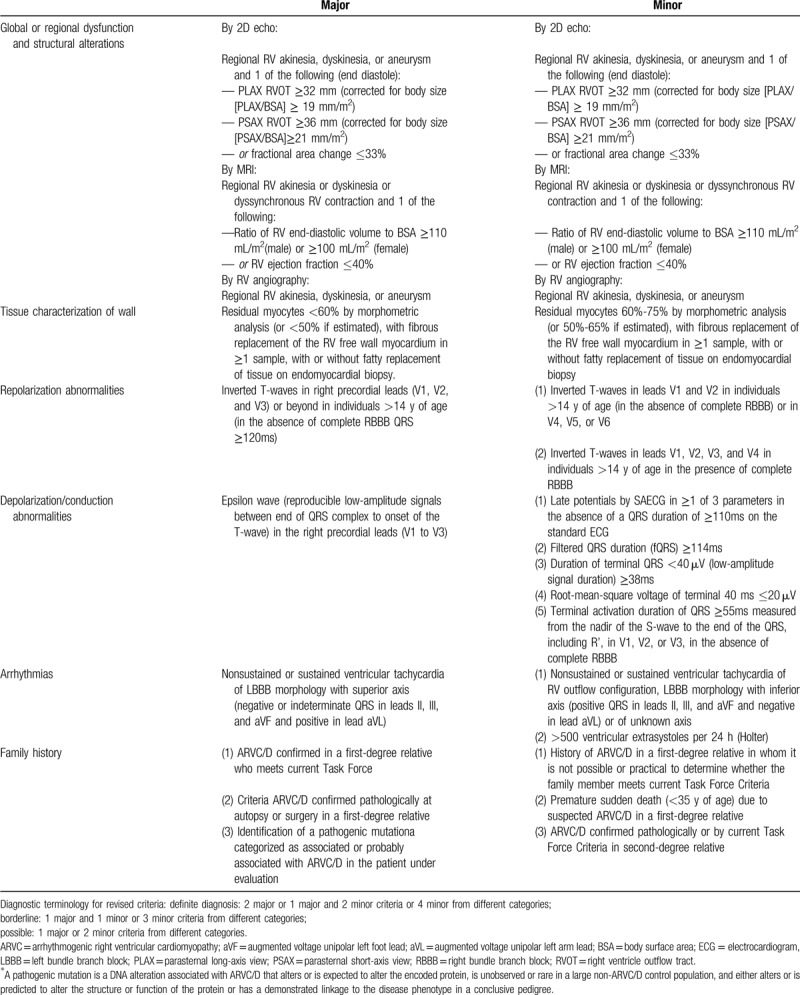
2010 Revised Task Force Criteria for the diagnosis of arrhythmogenic right ventricular cardiomyopathy.

In the case reported in this article, the patient had a large ASD. However, the patient's clinical presentation and accessory examination results seemed to also meet the 2010 Revised Task Force Criteria for the diagnosis of arrhythmogenic right ventricular cardiomyopathy. Can CHD such as ASD appear accompanied by ARVC? We reviewed the case of CHD combined with arrhythmogenic right ventricular cardiomyopathy reported since the concept of arrhythmogenic right ventricular cardiomyopathy was put forward. Consequently, we found that such cases were too scarce that only 5 cases were reported. The clinical and pathologic features of these cases are listed in Table 2.^[7–9]^ Through these cases, we can conclude that for the patients diagnosed with CHD, the clinical presentations of ARVC tend to be covered up. That is because ARVC and left-to-right shunting CHD at times share the common phenotype of right ventricle dysfunction despite different mechanisms of disease.^[9]^ The process of disease can be accelerated by conditions such as physical activities which disproportionately increase right ventricle stress.^[10]^ The study of La Gerche A et al^[11]^ showed that even for a healthy man, chronic right ventricle stress in an otherwise healthy heart could still result in a phenotype similar to ARVC in the absence of impaired desmosome. Thus, we can hypothesize that the chronic right ventricle volume overload secondary to left-to-right shunting CHD may cause structural and electrophysiologic findings to be consistent with those of ARVC. For one thing, the diagnosis of ARVC is challenging for young patients because absent phenotypic features and the overlapping of the findings with CHD are possible.^[9]^ For another, the symptoms of CHD patients sometimes meet the diagnosis criteria of ARVC regarding the secondary pathophysiologic change. Therefore, the early detection of CHD should be emphasized. Moreover, the cardiac MRI^[12]^ and gene detection may assist in the differential diagnosis.

**Table 2:**
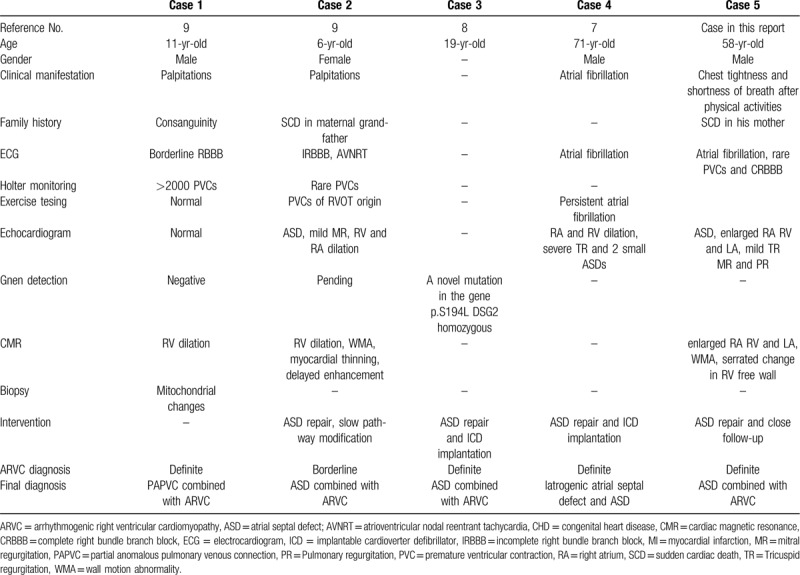
Clinical and pathologic features of 5 patients with ARVC versus CHD.

When it comes to the therapy, structural CHD may be managed with percutaneous or surgical interventions,^[13]^ and ARVC can be controlled by activity restriction, medical therapy, and antiarrhythmic therapies. Notably, an implantable cardioverter defibrillator should be placed when necessary.^[14,15]^ With appropriate management, mortality rates of arrhythmogenic right ventricular cardiomyopathy and CHD are both low. Therefore, careful evaluation and differentiation are of vital importance. For this patient, the results of his follow-up proved the effect of therapy. This conclusion illustrates that the treatment for CHD is useful for the relief of symptoms when the CHD is combined with ARVC. Moreover, further reexamination is still of vital importance.

The patient in this case is relatively elder among patients diagnosed with CHD combined with ARVC. And through the literature review, we summarized the similarities and differences between the 2 diseases and emphasize careful evaluation and differentiation. Close follow-up will be necessary for the patients. We hope that more accurate criteria and better therapy for similar cases could be put forward in the near future.

## 4 Statements

The video of the patient's echocardiography are provided in the supplemental videos.

Echocardiography1: The parasternal long axis view.

Echocardiography2: The apical four chamber view.

Echocardiography3: The subxiphoid four chamber view.

## Acknowledgment

We would like to acknowledge the librarians at the Libraries of Central South University for their efforts in obtaining primary resources for this case report and literature review. Additionally, we would like to acknowledge professors, colleagues, friends, and family members who assisted in the writing procedure of this case report.

## Author contributions

DR Chutong Ren and DR Jun Luo provided main efforts in the writing and analyzing procedure. Meanwhile, DR Yanshu Zhao and DR Zhenfei Fang as specialists in department of cardiology found the value of this case and provided great help in the diagnosing process.

**Funding acquisition:** Zhenfei Fang, Yanshu Zhao.

**Investigation:** Chutong Ren.

**Supervision:** Zhenfei Fang, Yanshu Zhao, Jun Luo.

**Writing – original draft:** Chutong Ren.

**Writing – review & editing:** Zhenfei Fang, Yanshu Zhao, Jun Luo.

## Supplementary Material

Supplemental Digital Content

## Supplementary Material

Supplemental Digital Content

## Supplementary Material

Supplemental Digital Content
